# Supplementary material for: Cardiac Tyrosine 97 Phosphorylation of Cytochrome c Regulates Respiration and Apoptosis
Source: Int J Mol Sci. 2025 Feb 4;26(3):1314. doi: 10.3390/ijms26031314 (PMC11818311; doi:10.3390/ijms26031314)
Supplement: Supplementary file 1 [file ijms-26-01314-s001.zip › Supplementary Figure S01 v02.pdf]

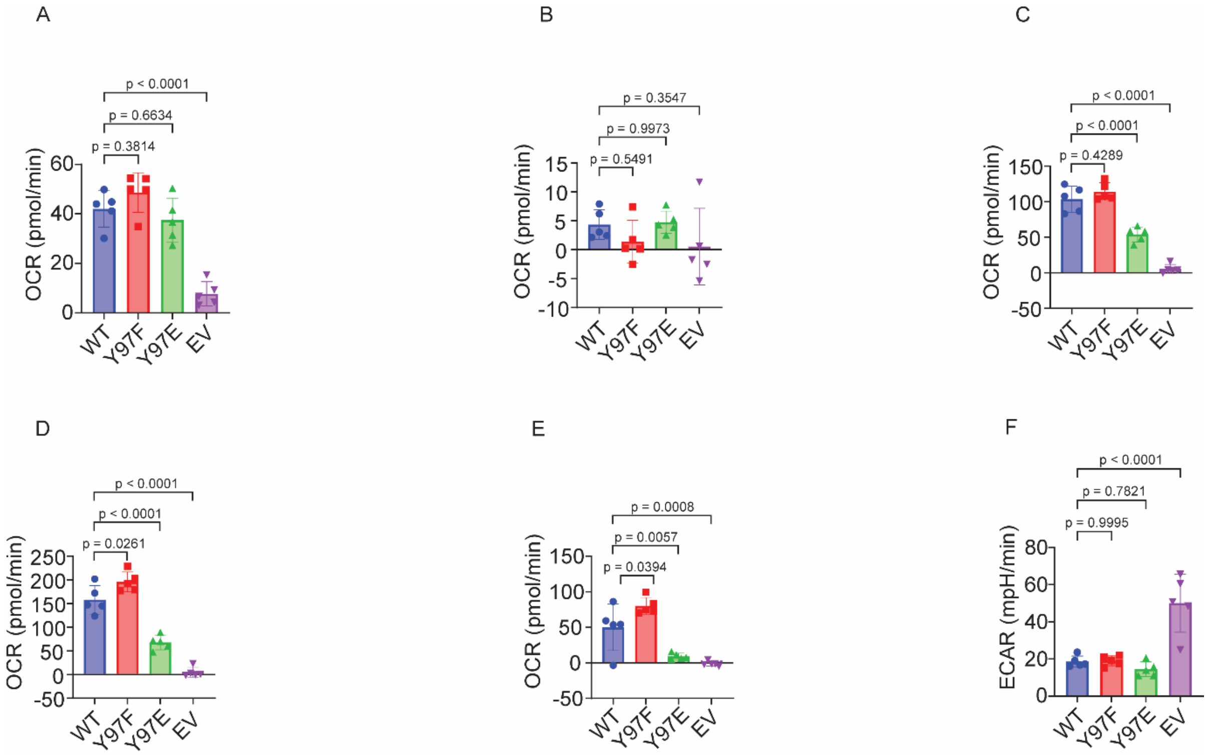


**Supplementary Figure S1.** All following figures are parameters calculated according to the manufacturer's protocol from the mitochondrial stress test shown in Figure 2B performed on a Seahorse bioanalyzer with sequential injections of 1 µM oligomycin, 2.5 µM carbonylcyanide-3-chlorophenylhydrazone, and 1 µM rotenone/antimycin A: **A** non-mitochondrial respiration (n = 5), **B** proton leak (n = 5), **C** ATP-coupled respiration (n = 5), **D** maximal respiration (n = 5), **E** spare respiratory capacity (n = 5), and **F** basal extracellular acidification rate (ECAR) (n = 5).
